# MiR-34b/c play a role in early sex differentiation of Amur sturgeon, *Acipenser schrenckii*

**DOI:** 10.1186/s12983-022-00469-6

**Published:** 2022-09-26

**Authors:** Xiujuan Zhang, Wenhua Wu, Jiabin Zhou, Linmiao Li, Haiying Jiang, Jinping Chen

**Affiliations:** 1 Guangdong Key Laboratory of Animal Conservation and Resource Utilization, Guangdong Public Laboratory of Wild Animal Conservation and Utilization, Institute of Zoology, Guangdong Academy of Sciences, Guangzhou, China; 2 Heilongjiang River Fisheries Research Institute, Chinese Academy of Fishery Sciences, Harbin, China

**Keywords:** Amur sturgeon (*Acipenser schrenckii*), miR-34b, miR-34c, Sex differentiation, Germ cell culture

## Abstract

**Background:**

Sex differentiation can be viewed as a controlled regulatory balance between sex differentiation-related mRNAs and post-transcriptional mechanisms mediated by non-coding RNAs. In mammals, increasing evidence has been reported regarding the importance of gonad-specific microRNAs (miRNAs) in sex differentiation. Although many fishes express a large number of gonadal miRNAs, the effects of these sex-biased miRNAs on sex differentiation in teleost fish remain unknown. Previous studies have shown the exclusive and sexually dimorphic expression of miR-34b/c in the gonads of the Amur sturgeon (*Acipenser schrenckii*), suggesting its potential role in the sex differentiation process.

**Results:**

Using quantitative real-time PCR (qPCR), we observed that miR-34b/c showed consistent spatiotemporal expression patterns; the expression levels significantly increased during early sex differentiation. Using in situ hybridization, miR-34c was found to be located in the germ cells. In primary germ cells *in vitro*, the group subjected to overexpression and inhibition of miR-34c showed significantly higher proliferation ability and lower apoptosis, respectively, compared to the corresponding control group. Luciferase reporter assays using the *ar*-3′UTR-psiCHECK-2 luciferase vector suggested a targeted regulatory interaction between miR-34b/c and the 3′UTR of the androgen receptor (*ar*) mRNA. Furthermore, miR-34b/c and *ar* showed negative expression patterns during early sex differentiation. Additionally, a negative feedback regulation pattern was observed between *foxl2* expression in the ovaries and *amh* and *sox9* expression in the testes during early sex differentiation.

**Conclusions:**

This study sheds new light on the roles of miR-34b/c in gonad development of Amur sturgeon, and provides the first comprehensive evidence that the gonad-predominant microRNAs may have a major role in sex differentiation in teleost fish.

**Supplementary Information:**

The online version contains supplementary material available at 10.1186/s12983-022-00469-6.

## Background

Sex determination and differentiation can be viewed as a complex genetic network of transcription factors that is initiated by a sex-determining switch mediating the expression of sex differentiation-related genes, thereby ultimately establishing and maintaining either the male or female phenotype [1]. The functions of many sex-associated genes appear to be relatively conserved during downstream sex differentiation processes in most vertebrates [1, 2]; these genes include *foxl2* (forkhead box L2) [3] and *dmrt1* (doublesex- and mab-3 related transcription factor 1) [4]. In some teleost fishes, *foxl2* and *dmrt1* show sex-dominant transcriptional expression in undifferentiated gonads during early ‘molecular sex differentiation’ [5]; subsequently, they show sexually dimorphic patterns, which play an essential role in subsequent ovarian differentiation or testis differentiation.

Differences between males and females with respect to the morphology, physiology, and behavior of gonochoristic teleost fishes are derived from sex-specific selection forces due to different genetic resources. However, except for the sex chromosome, both sexes share the same genome sequence; therefore, the characteristics and expression of sex-biased genes play a core role in shaping the phenotypic diversity of the two genders [6]. MicroRNAs (miRNAs) are endogenous, small, non-coding RNAs (19–25 nucleotides in length) that function as post-transcriptional repressors of gene expression by binding to complementary sites in the 3′-untranslated region (3′UTR) of target mRNAs. Increasing evidence has been reported regarding the importance of gonad-specific miRNAs in sex differentiation [7]. For example, in mice, miR-124 is involved in regulating the fate of developing ovarian cells by preventing the expression of *sox9* (SRY-box containing gene 9) [8]. Additionally, *sox9* regulates the expression of miR-202-5p/3p, a conserved miRNA that functions in the gonads during early testis differentiation [9]. Furthermore, the sexual regulator *dmrt1* has been identified as a direct target of miR-19a/b during the sex reversal process [10]. These studies show that sex differentiation in animals is regulated by numerous molecules and signaling networks at the transcriptional and post-transcriptional levels. In particular, appropriate regulation of sex differentiation-related gene expression results from a controlled balance in post-transcriptional mechanisms mediated by non-coding RNAs.

Sturgeons (Acipenseriformes) are an ancient fish group that originated during the Devonian period, over 200 million years ago; therefore, they constitute an ideal model species for studying the development and evolution of vertebrates [11, 12]. In addition to a teleost-specific round of whole-genome duplications (WGD) [13], Acipenseriformes experienced up to three lineage-specific WGDs [14, 15], resulting in the polyploidy patterns observed in sturgeons. Although sturgeons are dioecious, it is difficult to distinguish between females and males using morphological characteristics at the larval, juvenile, or even adult stages. The water temperature of the aquaculture does not affect the sex ratio (i.e., the sex differentiation of sturgeons shows no association with the breeding environment) [16]. Although the genome from two types of sturgeons has been decoded, no sex chromosomes have been found [17, 18]. The mechanism of sturgeon sex determination was speculated to involve multi-gene patterns; however, the regulatory mechanisms of sex differentiation in sturgeons remain poorly understood. Therefore, initiation of research on non-coding RNAs (including miRNA, piRNA, and lncRNA) of sturgeon gonads is an exciting development in this field; these studies may provide genetic resources for deeper investigation.

miR-34b and miR-34c, which originate from the miR-34b/c cluster and belong to the same family, share the same primary transcript and contain the same seed sequence. miR-34b/c plays an important role in testicular function in mammals. In mice, miR-34c is specifically and abundantly expressed in male germ cells [19], and miR-34b/c^−/−^ male mice can survive but cannot produce normal offspring. These models exhibit severely blocked testicular differentiation and reduced epididymis weight with abnormal spermatozoa and motility [20, 21]. miR-34b/c additionally plays a significant role in embryonic development. For example, high expression of miR-34c in human sperm cells was significantly correlated with the outcomes of intracytoplasmic sperm injection, including high embryo quality, implantation rate, pregnancy, and birth rate [22]. Increased miR-34c expression level in donor cells can significantly improve the early development of somatic cell nuclear transfer (SCNT) bovine embryos [23]. Meanwhile, low expression of miR-34b/c plays an irreplaceable role in maintaining normal ovarian function. For example, miR-34c with abnormal expression in ovarian cancer cell lines (SKOV3-ipl) has been reported to cause cell-cycle arrest in the G1 phase [24] and miR-34b/c has been shown to regulate the proliferation and apoptosis of ovarian surface epithelial cells [25]. In teleosts, the role of miR-34b/c in sex differentiation remains unclear.

We had previously screened the small RNA population of differentiated gonads in the Amur sturgeon (*Acipenser schrenckii*) using high-throughput sequencing to identify sex-biased miRNAs that may regulate early sex differentiation in sturgeons [26]. We determined that miR-34b/c was exclusively expressed in the gonads of juvenile sturgeons, and it exhibited sexually dimorphic expression patterns [27], which suggests that miR-34b/c is a candidate gene involved in the sex differentiation of sturgeons. To further characterize the role of miR-34b/c in regulating sex differentiation events in sturgeons, first, we investigated the expression levels and localization of miR-34b/c using quantitative real-time PCR and in situ hybridization during sex differentiation of *A. schrenckii*, which is a critically endangered and economically important aquaculture species [28]. Next, overexpression and inhibition experiments on miR-34b/c were performed in primary germ cells isolated and cultured *in vitro* from undifferentiated gonad tissues. Furthermore, we verified that the androgen receptor (*ar*) was likely to be the direct post-transcriptional target mRNA of miR-34b/c and that the expression patterns of seven sex-related mRNAs during sex differentiation were associated with the role of miR-34b/c.

## Materials and methods

### Experimental animals and ethical statement

We studied Amur sturgeon samples from five developmental stages during early sex differentiation: undifferentiated gonads (UGs; Amur sturgeons at 5 months after hatching (5 M) (N = 22) and differentiated gonads from four stages (testes and ovaries; 8 M (N = 30), 12 M (N = 30), 24 M (N = 7), and 36 M (N = 30)). All objectives were obtained from the Engineering and Technology Center of Sturgeon Breeding and Cultivation of the Chinese Academy of Fishery Science (Beijing, China). The growth performance of Amur sturgeon individuals is summarized in Additional file 1: Table S1.

All experimental Amur sturgeon individuals were anesthetized with 10^−4^ (v/v) eugenol in water for 1–3 min, following the AVMA guidelines (2013). In this study, gonad-tissue samples were prepared using three methods: (1) Bouin’s fixed gonads for further histological procedures and in situ hybridization (ISH) analysis, (2) immediate liquid nitrogen preservation until total RNA extraction, and (3) single-cell isolation of undifferentiated gonad tissues for germ cell culture in a sterile environment.

### Histological observation of gonads during sex differentiation

Gonads from the above five developmental stages were fixed in Bouin’s solution (prepared with 0.1% DEPC-treated water) for 20 h and transferred to 70% ethanol (prepared with 0.1% DEPC-treated water) for a longer period. Subsequently, the fixed gonads were dehydrated in an ascending series of graded ethanol concentrations and embedded in paraffin. Furthermore, cross-sections of 5 µm thickness were prepared, and histological analysis of each gonad tissue was performed using hematoxylin and eosin (HE) staining. The sections were observed and photographed using an EVOS FL Auto microscope and the corresponding cell imaging system (Thermo Fisher).

### miRNA in situ hybridization (miISH)

In situ hybridization experiments were performed for detecting miR-34b/c localization in the differentiated gonad specimens. Digoxigenin (DIG)-labeled locked nucleic acid probes of miR-34b-specific and miR-34c-specific were synthesized by Guangzhou Exonbio Inc (miR-34b probe: 5′-CAATCAGCTAACAACACTGCCTA-3′ and miR-34c probe: 5′- GTAATCAACTAACTGCACTGCCT-3′, labeled with Digoxin at 5′ and 3′). Gonads from Amur sturgeon individuals aged 36 M were further subjected to miISH analysis according to the method of enhanced sensitive ISH detection kit I (POD) (Boster, Wuhan, China). Briefly, Fresh and 5 µm thick paraffin sections were treated with a conventional dewaxing and rehydrate procedure. After incubating slides with 0.25% pepsin in 37 °C for 30 min, the slides were washed in 0.5 M PBS for 5 min, three times. Then the slides were treated with 3% H2O2 at room temperature for 30 min to ride off signal interference of endogenous peroxidase. After pre-hybridization with sufficient RNA hybridization buffer to each slide at 40 °C for 3 h, the slides were hybridized with DIG-labeled LNA probes diluted to 1:100 with a miRNA hybridization buffer overnight at 40 °C in a moist chamber. By submerging the slides in 0.5 M PBS/0.7‰ tween 20 and washing for 15 min, three times. Signals were then detected using anti-digoxigenin -conjugated antibodies in 37 °C for 1 h and SABC-POD solution in 37 °C for 20 min. Positive results were visualized using 3,3′-diaminobenzidine (DAB) staining and cell nuclear staining of hematoxylin was also performed. Meanwhile, negative controls of above miISH were designed for hybridization with blank miRNA hybridization buffer.

### RNA extraction and quality evaluation

Total RNA was extracted from each gonad sample using the RNAiso reagent (TaKaRa, Tokyo, Japan). RNA purity was detected using 1.5% gel electrophoresis. RNA concentration and purity were measured using a microplate reader (Thermo Fisher Scientific). The RNA quality criteria for clear bands of 28S and 18S rRNA and OD 260/280 > 1.8 were used. Qualified RNAs were used for further expression assays using quantitative real-time PCR and RT-PCR.

### Quantitative real-time PCR assay

miR-34b (5′-UAGGCAGUGUUGUUAGCUGAUUG-3′) and miR-34c (5′-AGGCAGUGCAGUUAGUUGAUUAC-3′) expression was examined using stem-loop reverse transcription (RT) real-time PCR. The spatiotemporal expression patterns of three ovary differentiation-related genes (*foxl2*, *er*, and *cyp19a*) and four testis differentiation-related genes (*amh*, *ar*, *sox9*, and *dmrt1*) in the gonads were analyzed using quantitative real-time PCR. Gonads from four developmental stages were considered, ranging from undifferentiated gonads (UGs; Amur sturgeons at 5 M, N = 4) to differentiated gonads (12 M, 24 M, and 36 M, every stage includes 3 testes and 3 ovaries).

Briefly, 200 ng of total RNA from each sample was reverse-transcribed using the First-strand cDNA Synthesis Kit (TaKaRa, Tokyo, Japan) with stem-loop RT primers and oligo(dT)18 primers designed according to the methods described by Chen et al. [29]. Reactions were allowed to proceed at 42 °C for 60 min and 70 °C for 15 min and subsequently held at 4 °C. Blank and template-free reactions served as negative controls. The primers of the sex differentiation-related and housekeeping genes were designed using Primer Premier 6 software. The sequences information of the stem-loop RT primers for miR-34b/c and primers of sex differentiation-related and housekeeping (U6 snRNA or *β-actin*) genes including with the Genbank accession numbers of NCBI database, are listed in Additional file 2: Table S2. Real-time PCR was performed using the Applied Biosystems Quant-Studio™ 5 platform (Thermo Fisher Scientific) according to the manufacturer's protocol. A total of 0.5 μL of cDNA was used as the template in a 20 μL reaction mixture, along with 10 μL of the SYBR® Green Master Mix (Applied Biosystems, Carlsbad, USA), 0.5 μL of each primer (10 μM), and 8.5 μL of ultrapure water under the following conditions: 50 °C for 2 min for Heated-labile Uracil-DNA Glycosylase (UDG) activation and subsequently 95 °C for 2 min, followed by 40 cycles of 95 °C for 15 s, 60 °C for 30 s, and 72 °C for 30 s.

Each targeted gene was analyzed in triplicate in more than three Amur sturgeon individuals (biological replicates). The expression levels of the targeted genes were calculated using the relative quantity (2^−△△CT^) method after normalization against the U6 snRNA or *β-actin* gene and UGs (undifferentiated gonads, i.e. 5 M) as reference samples.

### Isolation, *in vitro* culture, and identification of gonadal germ cells from *A. schrenckii*

Single germ cells were isolated from the gonads of Amur sturgeons at the sensitive stage of early sex differentiation, ranging from 5 to 7 M. Gonadal tissues are surrounded by thick yellow-white fat, which significantly increases the difficulty of separation. Therefore, after removal of as much fat as possible from gonadal tissues, single germ cells were successfully isolated using a one-step digestion method with a three-enzyme mixture at relatively lower temperatures. Briefly, 1) the gonadal fat was carefully removed under a stereoscopic microscope in a sterile room, and the clean gonad tissue was transferred to a new Petri dish containing PBS solution and washed three times; 2) the gonad tissues were cut into pieces as small as possible and transferred to a 15 mL sterile centrifuge tube filled with a mixed-enzyme digestion juice (containing 1.5 mg/mL type IV collagenase, 20 µg/mL DNA enzyme I, and 0.25% trypsin–EDTA solution) of 10 times volume in a 25 °C water bath for 2.5–3.5 h; 3) the mixed digestion was then terminated with an equal volume of Dulbecco’s Modified Eagle Medium (DMEM) with 10% fetal calf serum (FCS), and undigested tissues were filtered using a 100 µm cell sieve. The cell viability was shown to be more than 95% by the trypan blue exclusion method.

The complete culture medium consisted of MEMα (Gibco) and 10% FCS (Gibco). Four growth factors were added for the renewal and maintenance of undifferentiated germ cells, including 10 ng/mL LIF (Peprotech), 100 ng/mL EGF (Peprotech), 20 ng/mL GDNF (Peprotech), and 10 ng/mL bFGF (Peprotech). The culture conditions were 25 °C and 5% CO_2_.

We identified seven germ cell-specific marker genes in the cultured germ cells, ovaries, and testes from Amur sturgeon individuals at 36 M; these markers were five undifferentiated markers (*dead* end (*dnd1*), glutamate receptor interacting protein 2 (*grip2*), Nanog, Deleted in azoospermia-like (*dazl*)) and two differentiated markers (synaptonemal complex protein 3 (*scyp3*) and zona pellucida 3 (*zp3*)). The primer sequences for these seven genes are listed in Additional file 3: Table S3.

### miR-34b/c mimic and miR-34b/c inhibitor transfected cultured germ cells *in vitro*

On the fifth day of the *in vitro* culture, the germ cells began to proliferate, whereas somatic cells showed no significant change because of contact growth inhibition as feeder layer cells. Therefore, primary germ cell cultures on the fifth day *in vitro* were selected for transfection for miRNA overexpression and inhibition experiments. Cells were plated at a density of 1 × 10^4^–2 × 10^4^ cells in a 96-well Petri dish with 200 µL of complete culture medium. Custom miR-34b/c mimics or miRNA inhibitors synthesized using the mature sequence of miR-34b/c (RiboBio, Guangzhou, China) were used. According to the guidelines of the manufacturer, after preparing with a mixture of 0.5 µL of Lipofectamine 2000 (Invitrogen, CA, USA) in 25 µL of Opti-Men-I reduced serum medium without antibiotics and 0.5 µL of miRNA mimic or miRNA inhibitors or NC control (50 nM) in 25 µL of Opti-Men, primary germ cells per wells on the fifth day of *in vitro* culture were transfected for 4–6 h. After transfection, the complete culture medium containing serum and antibiotics was replaced, and the cultures were continued for 48–72 h. The three independent transfected experiments were performed.

### Cells proliferation and apoptosis assay

The proliferation activity of transfected cells was detected using the CellTiter 96^®^ AQ_ueous_ non-radioactive Cell Proliferation Assay (MTS) (Promega), and the absorbance values at 490 nm were analyzed using a Multiskan™ FC instrument (Thermo Fisher).

Subsequently, the apoptosis of cultured germ cells was examined by Annexin V-FITC/PI double labeling method with the Annexin V-FITC Apoptosis Kit (Invitrogen, CA, USA, BMS500FI-100) according to the manufacturer’s instructions. Briefly, (1) According to the growth characteristics *in vitro* of germ cells, the attached germ cells were harvested as much as possible using short time digestion method with 0.25% trypsin–EDTA solution under 25 °C incubator. (2) The harvested cells were washed in PBS two times, and then were resuspended in 200 µL Bing buffer (1 ×). (3) Resuspended cells were stained with Annexin V-FITC and 20 µg/ml Propidum lodide (PI) in the dark, respectively. The treated cells of different groups were respectively observed and photographed using an inverted fluorescence microscope and the corresponding cell imaging system (Olympus IX71). Apoptosis analysis was performed by fluorescence microscopy, and calculated by the ratio of positive-staining cells to the total cells in same field.

### miRNA target prediction

miR-34b and miR-34c, which belong to the same family with the same seed sequence, are important candidate genes that are differentially expressed in the ovaries and testes of juvenile Amur sturgeons [27]. Using the computational prediction of microRNA/target duplexes RNA software RNAhybrid [30] (https://bibiserv.cebitec.uni-bielefeld.de/rnahybrid), 3′UTR of the *ar* gene and the seed sequence of miR-34b and miR-34c were predicted to exhibit possible regulation site pairs.

### Construction of *ar*-3′UTR-psiCHECK-2 luciferase vector

KOD_Plus-Neo high-fidelity PCR enzyme (Toyobo) was used in a 20 µL reaction mixture for amplifying the wild-type 3′-UTR region of *ar*. Information on primers containing XhoI and NotI restriction sites is included in Additional file 4: Table S4. Subsequently, a total of 70 µL of double enzyme digestion system mixture were used for obtaining the target fragment; the mixture included 20 µL of cDNA, 6 µL of 10 × Buffer O, 4 µL of Not I (Thermo Fisher), 8 µL of Xhol (Thermo Fisher), and ddH_2_O. The reaction program settings were 37 °C, 16 h; 80 °C, 20 min. Following the reaction, gel electrophoresis was used for evaluating the results of the double enzyme digestion. Subsequently, the PsiCHECK-2 luciferase vector (Promega) cut by the corresponding enzymes (i.e., XhoI and NotI) and *ar*-3′UTR target fragments were connected according to the instructions of the T4 DNA linking kit (Thermo Fisher). The plasmid of the *ar*-3′UTR-psiCHECK-2 expression vector was extracted according to the instructions of the Endo-free Plasmid Mini Kit II (OMEGA).

### Luciferase assay identification

HEK-293 T cells were cultured in a 96-well Petri dish, and each well was transfected with 100 ng of the *ar*-3′UTR-psiCHECK-2 plasmid with 50 nM miR-34b mimics, miR-34c mimics, miR-34b + miR-34c mimics, or mimic NC control, with a mixture of Lipofectamine 2000 (Invitrogen, CA, USA) and OptiMEM (Invitrogen, CA, USA) without antibiotics, according to the manufacturer’s protocol. After 4–6 h of transfection, the complete culture medium containing serum and antibiotics was replaced, and the cultures were continued for 48–72 h. Subsequently, the cells were harvested and assayed using the Dual-Glo Luciferase Assay System (Promega), and the Synergy 2 multifunctional plate detector (BioTek) was used for measuring firefly and Renilla fluorescence, respectively. The firefly/Renilla luciferase activity ratio were respectively counted from three experimental groups and the corresponding NC control group.

### Statistical analysis

The data are expressed as the mean ± standard deviation of the measurements (M ± SD). Statistical analyses were performed using an independent samples *t*-test in SPSS 17.0. *P* < 0.05 was considered to indicate a statistically significant difference.

## Results

### Morphological changes during sex differentiation of the gonads in *A. schrenckii*

**Fig. 1:**
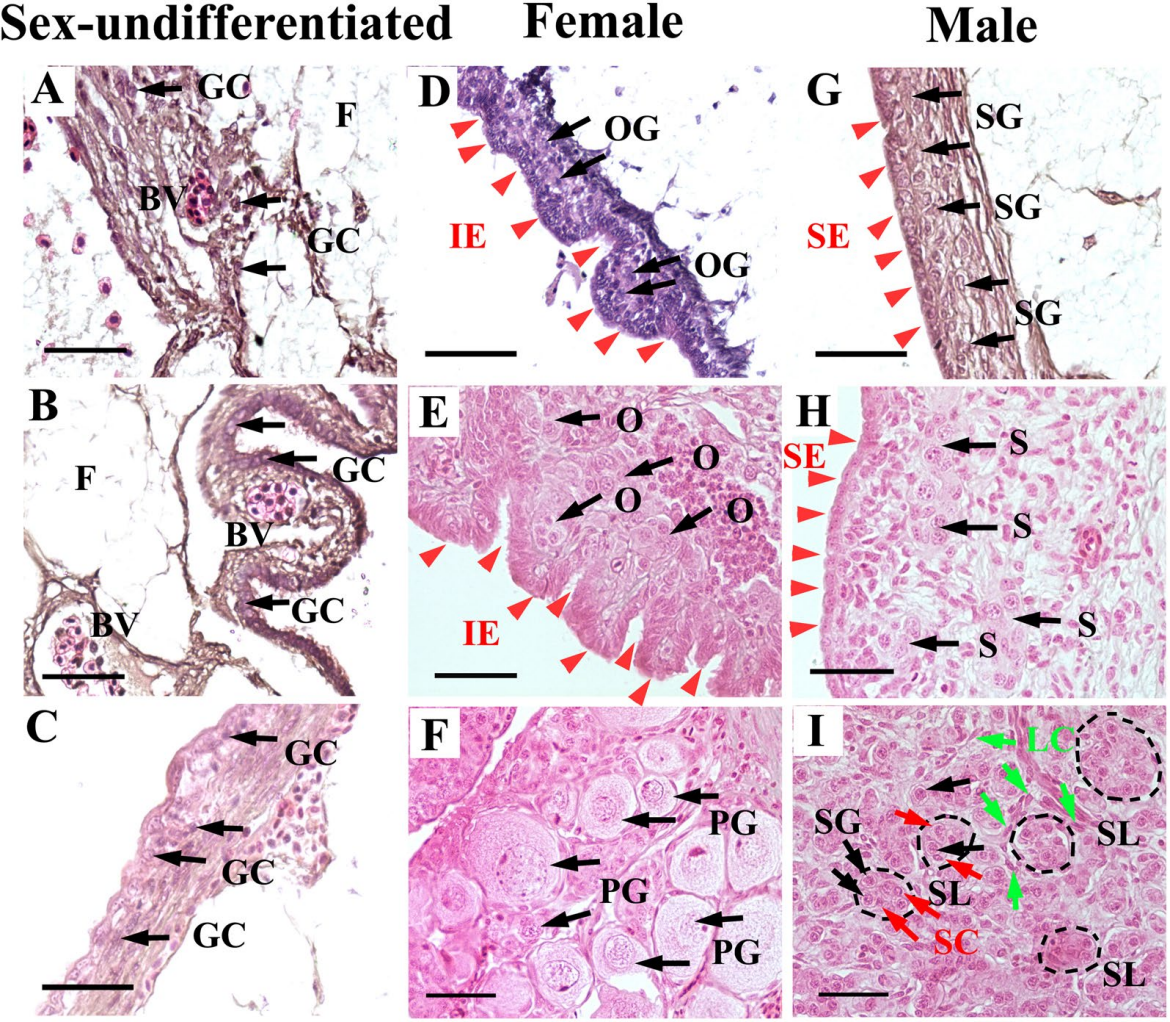
The histomorphological features and germ cell developmental patterns of gonads from Amur sturgeons during early sex differentiation. The gonads were at sex-undifferentiated stages (**A**, **B** 5 months after hatching (M); **C** 6 M). The surface of the gonad tissue was covered with a large amount of fat with continuous or discontinuous distribution. Ovarian differentiation was first recognizable by 8 M, which contained a folded or invaginated epithelium (IE) with germ cells (oogonia) underneath (**D**). The invaginated epithelium deepened and contained a few clusters of oogonia at 12 M (**E**) and primary growth oocytes at 24 M (**F**). Testis differentiation features were evident with a smooth epithelium (SE) with germ cells (spermatogonia) by 8 M (**G**), containing a few clusters of spermatogonia by 12 M (**H**), and presenting typically obvious seminal lobules at 24 M (**I**). BV, blood vessels; F, fat; GC, gonocyte; IE, invaginated epithelium; SE, smooth epithelium; OG, oogonia; SG, spermatogonia; O, cluster of oogonia; S, cluster of spermatogonia; PG, primary growth oocyte; SL, seminal lobules; SC, Sertoli cell; LC, Leydig cell; The gonadal tissues were stained with hematoxylin and eosin (HE staining). Scale bar = 50 μm

**Fig. 2:**
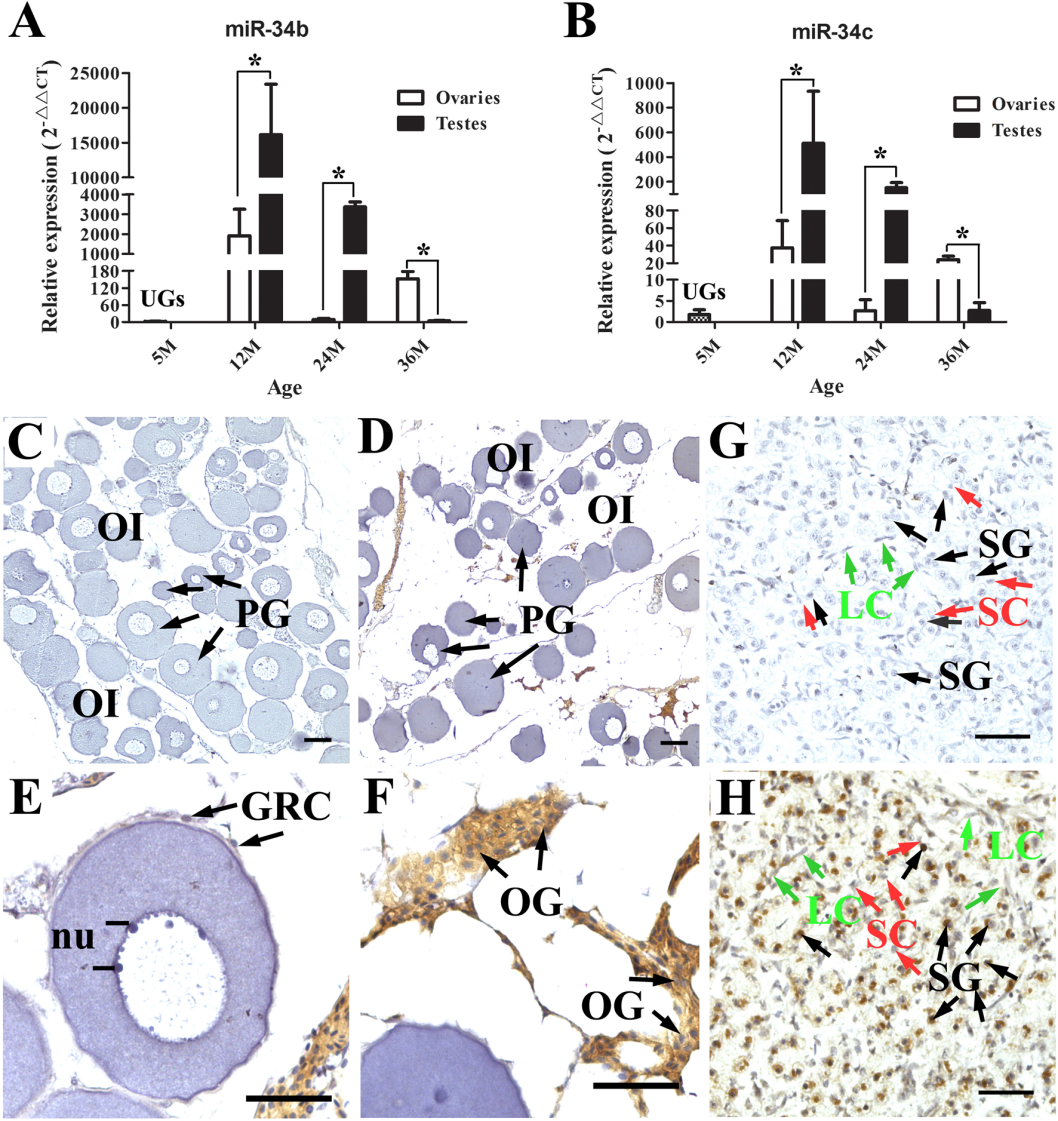
miR-34b/c expression in gonads during sex differentiation of *A. schrenckii.* **A**, **B** Spatiotemporal expression patterns of miR-34b and miR-34c using real-time PCR. The relative expression levels of miR-34b/c were normalized against the UGs (undifferentiated gonads, i.e. 5 M) as reference samples. *Represents *P* < 0.05 with a statistically significant difference between the two groups. Subsequently, location expression of miR-34c were detected in ovaries (**C**–**F**) and testes (**G**, **H**) of 36 M Amur sturgeon individuals using in situ hybridization (miISH). **C**, **G** were negative controls detected for hybridization with blank miRNA hybridization buffer. OI, ovarian lamellae; PG, primary growth oocyte; nu, oocyte nucleoli; GRC, granulosa cell; OG, oogonia; SG, spermatogonia; SC, Sertoli cell; LC, leydig cell.. Scale bar in C and D = 100 μm and in E, F, G and H = 50 μm

To investigate the role of miR-34b/c during sex differentiation, we first observed histomorphological features and germ cell developmental patterns. HE staining was used to study the gonadal tissue differentiation characteristics of Amur sturgeons during the developmental stages, ranging from 5 to 36 months after hatching (M). The gonad tissues from 5 to 6 M were in a sex-undifferentiated state filled with blood cells; however, no morphological differences were observed in all individuals (Fig. 1A–C). Meanwhile, the surface of the gonad tissue was covered with a large amount of fat with continuous or discontinuous distribution; therefore, the volumes were relatively low and the number of germ cells was few. The gonad volumes at 6 M were significantly larger than that at 5 M (Additional file 5: Fig. S1). The typical germinal epithelium formed gradually, and the number of germ cells with large cell nuclei increased significantly at 6 months (Fig. 1C). Morphological sex differentiation was evident until 8 M. At the same time, three types of gonadal features were observed: (1) the gonads showed a folded or invaginated epithelium with germ cells (oogonia) underneath, a sign of ovary differentiation (Fig. 1D); (2) the gonads showed a smooth epithelium with germ cells (spermatogonia), a sign of testis differentiation (Fig. 1G); and (3) the germinal epithelium of a few gonads were indistinguishable, and they appeared to be in a sex-undifferentiated state. At 12 M, the folds of the germinal epithelium of the ovary deepened and it contained a few clusters of oogonia (Fig. 1E). Meanwhile, the germ cells of the early testis were mainly at the stage of type A single spermatogonial cells (single) and occasionally at the stage of proliferating chains of paired spermatogonial cells (paired) (Fig. 1H). Seminal lobules in the testes and early oviposition plates in the ovaries were formed at 16 M. Meanwhile, all Amur sturgeon individuals completed morphological sex differentiation by 16 M. The most striking characteristic of the ovary at 24 M was developmental asynchronism. For example, some ovaries were mainly clusters of oogonia, and some ovaries began to appear a small collection of primary ovarian oocytes; however, some ovaries had been in the majority of growth oocytes (diameter approximately 50 μm) (Fig. 1F). A section of the testis at 24 M showed more obvious seminal lobules and significantly increased undifferentiated spermatogonial cells (Fig. 1I). Morphological features of the ovaries and testes at 36 M have been previously described [31].

### miR-34b/c expression in gonads during sex differentiation of *A. schrenckii*

Our previous study indicated that miR-34b and miR-34c were exclusively expressed in the gonads and sexually dimorphic in juvenile Amur sturgeons aged 11 M, which strongly implies their important roles in gonad differentiation [27]. Furthermore, spatiotemporal expression patterns in gonad-only tissues during sex differentiation were investigated using real-time PCR. The results of real-time PCR indicated that miR-34b and miR-34c exhibited similar spatiotemporal expression patterns (Fig. 2A, B). The expression levels of miR-34b and miR-34c were extremely low in the UGs, i.e., at 5 M; subsequently, they significantly increased with early sex differentiation. Among all examined developmental stages, the expression levels of miR-34b and miR-34c were the highest in the gonads at 12 M for both sexes and were significantly higher in the testes than in the ovaries from 12 to 24 M (*P* < 0.05). Subsequently, at 36 M, the expression pattern of miR-34b and miR-34c showed a dramatic change; the expression levels in the ovaries were significantly higher than that in the testes (*P* < 0.05). To determine the regions of expression, we further performed ISH detection of miR-34c using an enhanced and sensitive method in gonads at 36 M. In the ovary, miR-34c was mainly expressed in the cytoplasm of oogonia cells but not in the growth oocytes (Fig. 2C–F). Meanwhile, miR-34c was located in the nucleus of spermatogonial cells of the testis but not in somatic cells such as Leydig cells and Sertoli cells (Fig. 2G, H).

### Effect of miR-34b/c on the proliferation of gonad germ cells *in vitro*

To investigate the effect of miR-34b/c on germ cells in an *in vitro* culture, we attempted to establish an *in vitro* primary culture system of germ cells isolated from undifferentiated gonads of Amur sturgeon individuals (aged 5 M to 7 M). The culture proliferation characteristics of germ cells were stable after over three independent cell culture experiments (Additional file 6: Fig. S2A-D). After 48 h, fat cells and somatic cells were attached to the bottom of the culture plate, forming a layer of “feeder cells,” and single or multiple germ cells aggregated on the feeding layer into three-dimensional (3D) suspension growth. Small clonal clusters of 2–3 germ cells were occasionally observed. After 4 days, the germ cells began to proliferate, their numbers increasing significantly; but there was no remarkable change owing to contact inhibition of “feeder cells”. After 7 days *in vitro*, the germ cells continued to proliferate and formed larger clonal clusters. Cultured germ cells expressed five undifferentiated germ cell markers (*vasa*, *dnd1*, *grip2*, *nanog*, and *dazl*) but did not express two differentiated markers (*scyp3* and *zp3*) (Additional file 6: Fig. S2E). Thus, we successfully established a culture system of the primary germ cells of the Amur sturgeon.

**Fig. 3:**
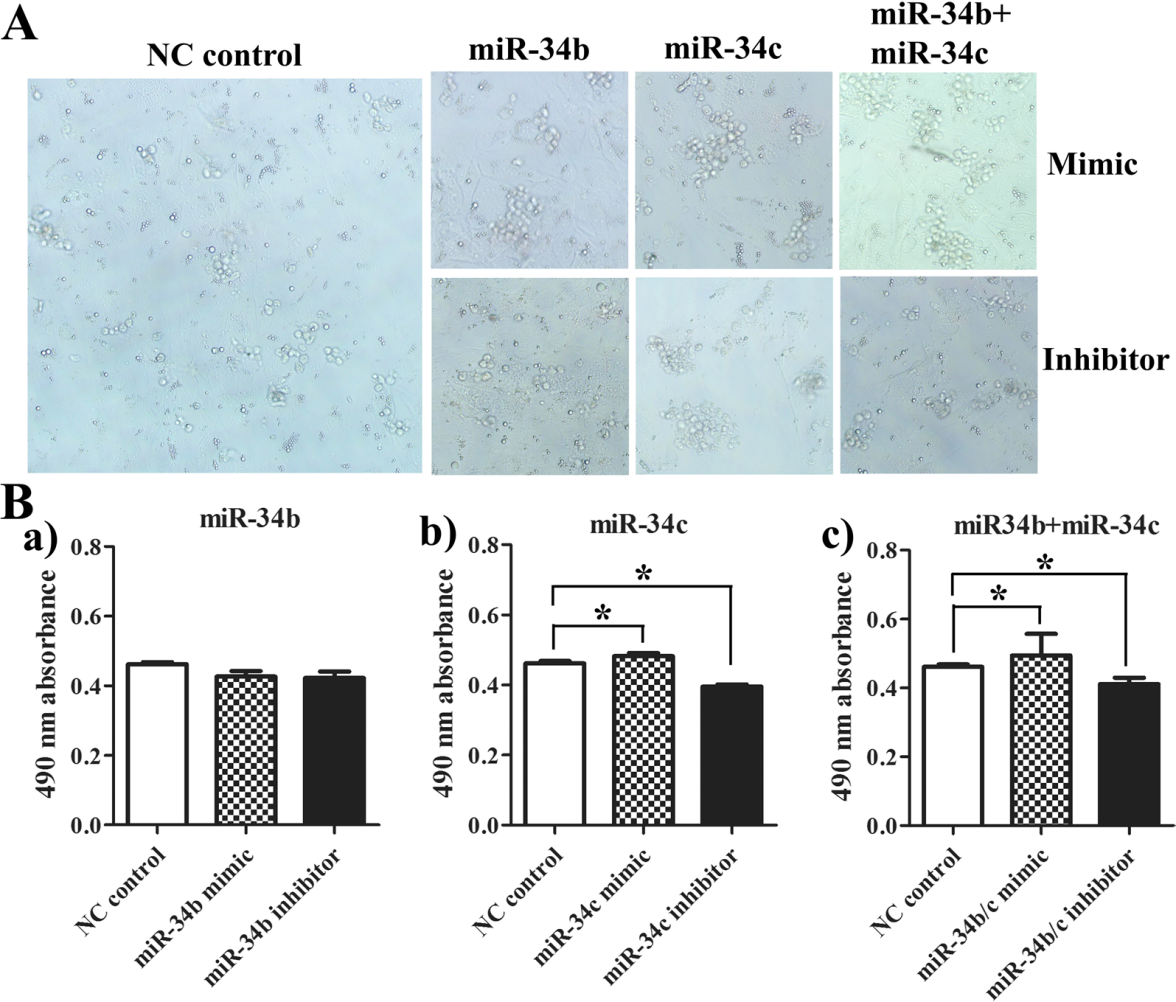
The effects of miR-34b/c mimic and inhibitor on proliferation of cultured germ cells *in vitro* of *A. schrenckii.* **A** The growth status of primary germ cells in different treatment groups. **B** The cell proliferation assay in the miR-34b groups (Ba), miR-34c groups (Bb), and miR-34b + miR-34c groups (Bc), respectively. *Represents *P* < 0.05 with a statistically significant difference between the two groups

On the fifth day of the *in vitro* culture, the germ cells began to proliferate, and somatic cells showed no significant change owing to contact growth inhibition as feeder cells. Therefore, primary germ cell cultures on the fifth day *in vitro* were selected for transfection in miRNA overexpression and inhibition experiments. The results showed that the overexpression of miR-34c (mimic group) and the co-overexpression of joint miR-34b and miR-34c (mimic group) significantly promoted the proliferation of primary germ cells compared with their NC control group (*P* < 0.05) (Fig. 3A, Ba–c). As expected, all inhibitor groups reduced the number of primary germ cells compared to the NC control group (Fig. 3A, Ba–c).

### Effect of miR-34b/c on the apoptosis of gonad germ cells *in vitro*

**Fig. 4:**
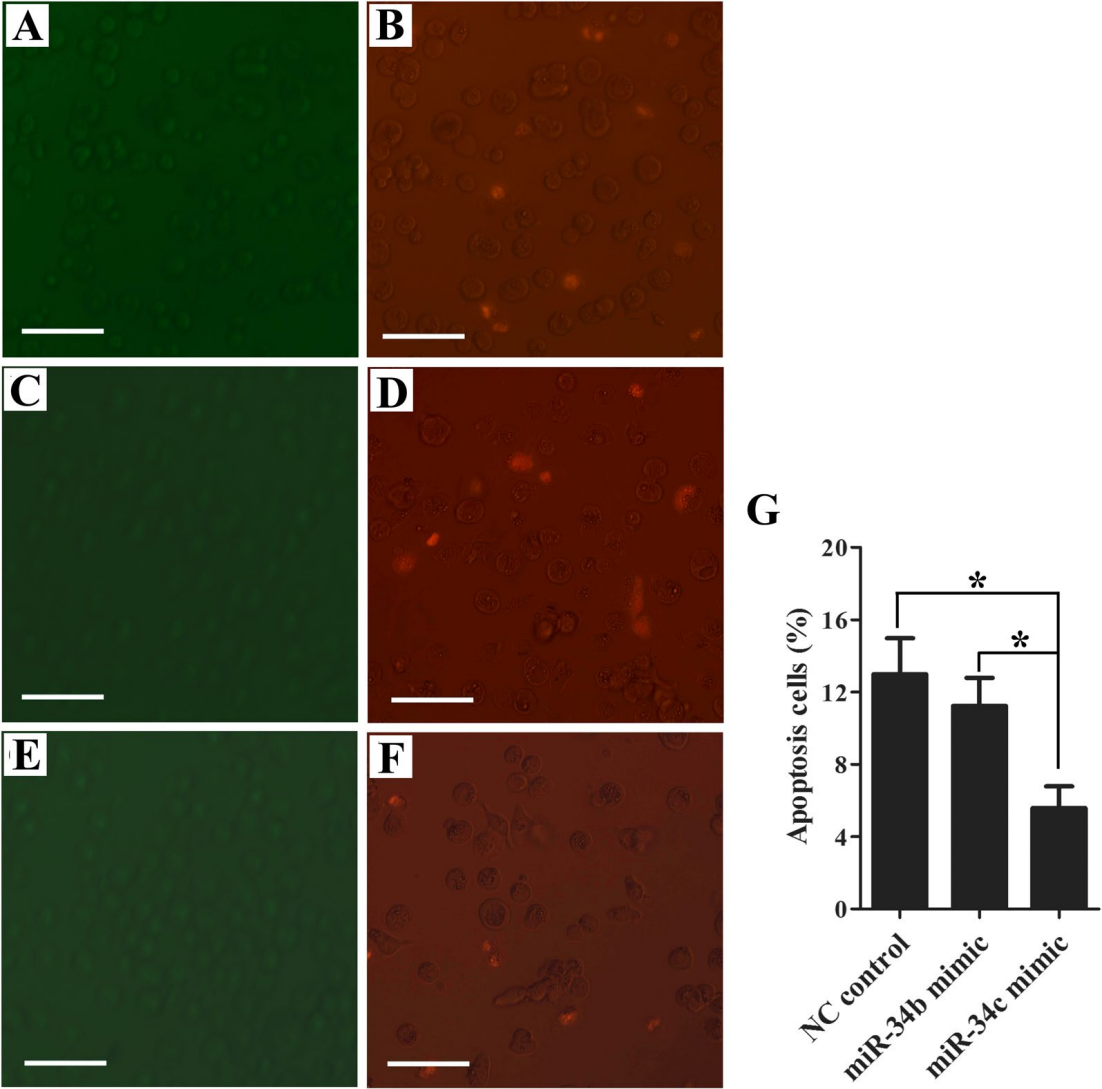
The effect of miR-34b and miR-34c mimics on apoptosis of cultured germ cells *in vitro* of *A. schrenckii*. Annexin V-FITC staining (green-fluorescence, **A**, **C**, **E**) stands for the early apoptotic cells and propidium iodide staining (PI, red-fluorescence, **B**, **D**, **F**) stands for the middle and late apoptotic cells.** A**–**F** represent the NC control, miR-34b and miR-34c mimic group, respectively. **G** The apoptosis of transfected germ cells detected by red fluorescence were counted in different groups. *Represents *P* < 0.05 with a statistically significant difference between the two groups. Scale bar = 50 μm

Annexin V-FITC staining was negative in both the experimental groups (miR-34b and miR-34c mimic groups) and the NC control group, which indicated that there were no effects on early apoptotic cells (Fig. 4A, C, E). Furthermore, according to the results of propidium iodide (PI) staining, the miR-34c overexpression group (Fig. 4F) exhibited a significantly reduced number of cells in the middle and late apoptotic cells compared with the NC control (Fig. 4B) (5.59% ± 2.40% vs. 12.97% ± 3.48%) and miR-34b-mimic groups (Fig. 4D) (5.59% ± 2.40% vs. 11.22% ± 3.49%) (*P* < 0.05) (Fig. 4G).

### miR-34b and miR-34c directly acted on the 3′UTR of ***ar*** mRNA

**Fig. 5:**
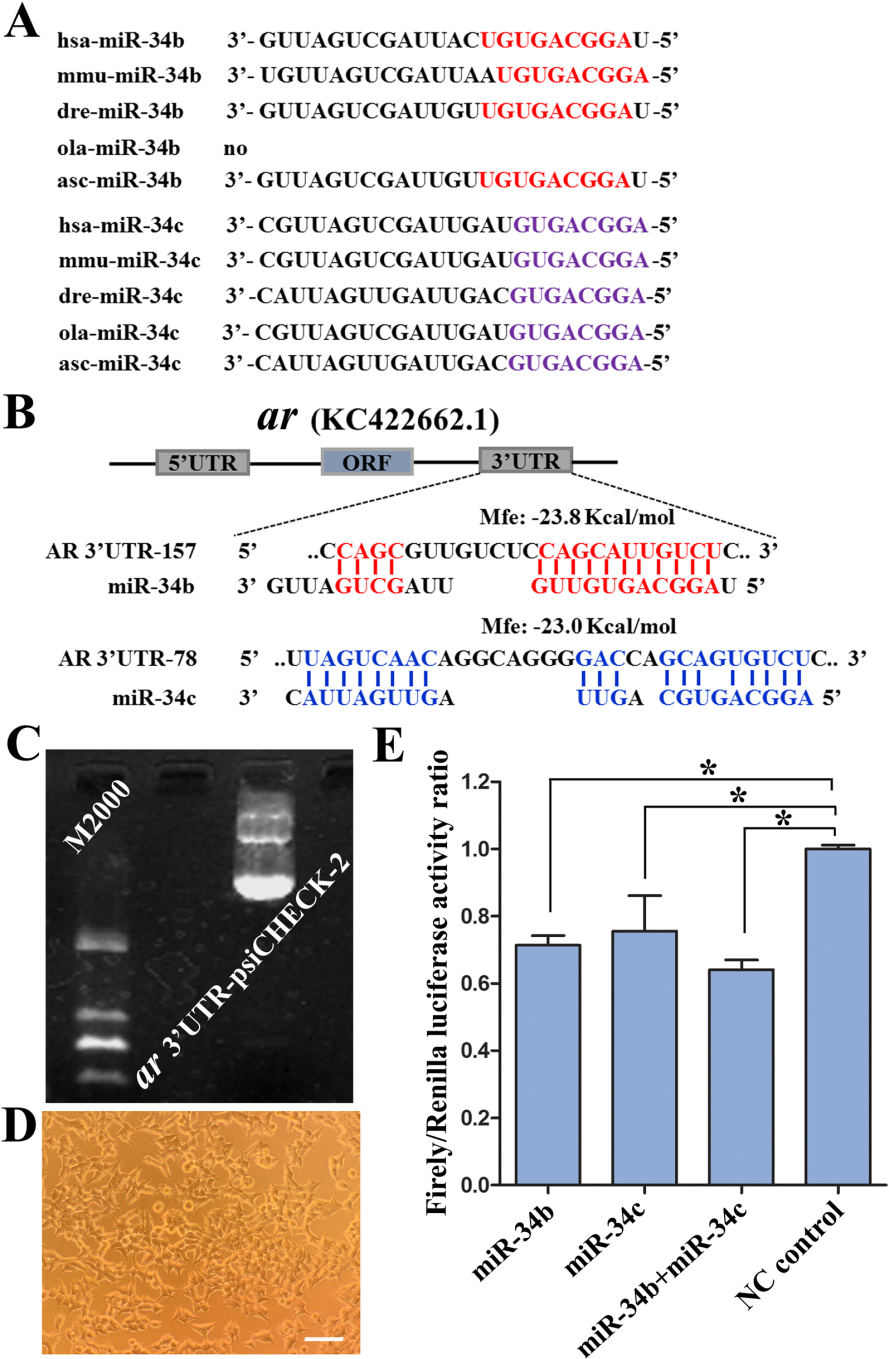
Sequences conservation analysis of miR-34b/c between different species and their targeted mRNA prediction and verification. **A** Sequences conservation analysis of miR-34b/c between different species. has, *Homo sapiens*; mmu, *Mus musculus*; dre, *Danio rerio*; ola, Oryzias latipes; asc, *Acipenser schrenckii*. **B** The relationship of miR-34b/c and the 3′UTR of *ar* mRNA was predicted with the RNAhybrid software. **C** The quality of plasmid extracted from *ar*-3′UTR-psiCHECK-2 luciferase vector was evaluated using gel electrophoresis. Marker 2000 was used. **D** The cultured HEK-293 T cells were co-infected with *ar*-3′UTR-psiCHECK-2 luciferase vectors and mimic of three experimental groups (miR-34b, miR-34c, and miR-34b + miR-34c) and the NC group (negative control), respectively. Scale bar = 50 μm. **E** The firefly/Renilla luciferase activity ratio were respectively counted from three experimental groups and the corresponding NC control groups. *Represents *P* < 0.05 with a statistically significant difference between the two groups

Sequences conservation analysis of miR-34b/c between different species indicated that the sequences of miR-34b/c from *A. schrenckii* were relatively conserved with zebrafish, medaka, mouse, even human, especially in the seed sequence (Fig. 5A). The sequences of miR-34b and miR-34c were hybridized to the best fitting part of the 3′UTR of *ar* mRNA with the minimum free energy hybridization using the RNAhybrid tool [30]. Among the predicted targets, *ar* was predicted to be a target gene of miR-34b and miR-34c (Fig. 5B). To determine whether miR-34b and miR-34c directly bind to the *ar* 3′UTR, we performed luciferase reporter assays by constructing the *ar*-3′UTR-psiCHECK-2 luciferase vector (Additional file 7: Fig. S3A-B). The corresponding results indicated that the miR-34b and miR-34c mimics significantly reduced *ar* 3′UTR-dependent firefly luciferase activity, respectively, whereas the NC control exhibited no effect on firefly luciferase activity. Meanwhile, the miR-34b + miR-34c mimic group also showed lower *ar* 3′UTR-dependent firefly luciferase activity (*P* < 0.05) (Fig. 5C–E). The above analysis suggested a targeted regulatory interaction between miR-34b and miR-34c and the 3′-UTR of *ar* mRNA.

### The expression of conserved sex-related genes during sex differentiation of *A. schrenckii*

**Fig. 6:**
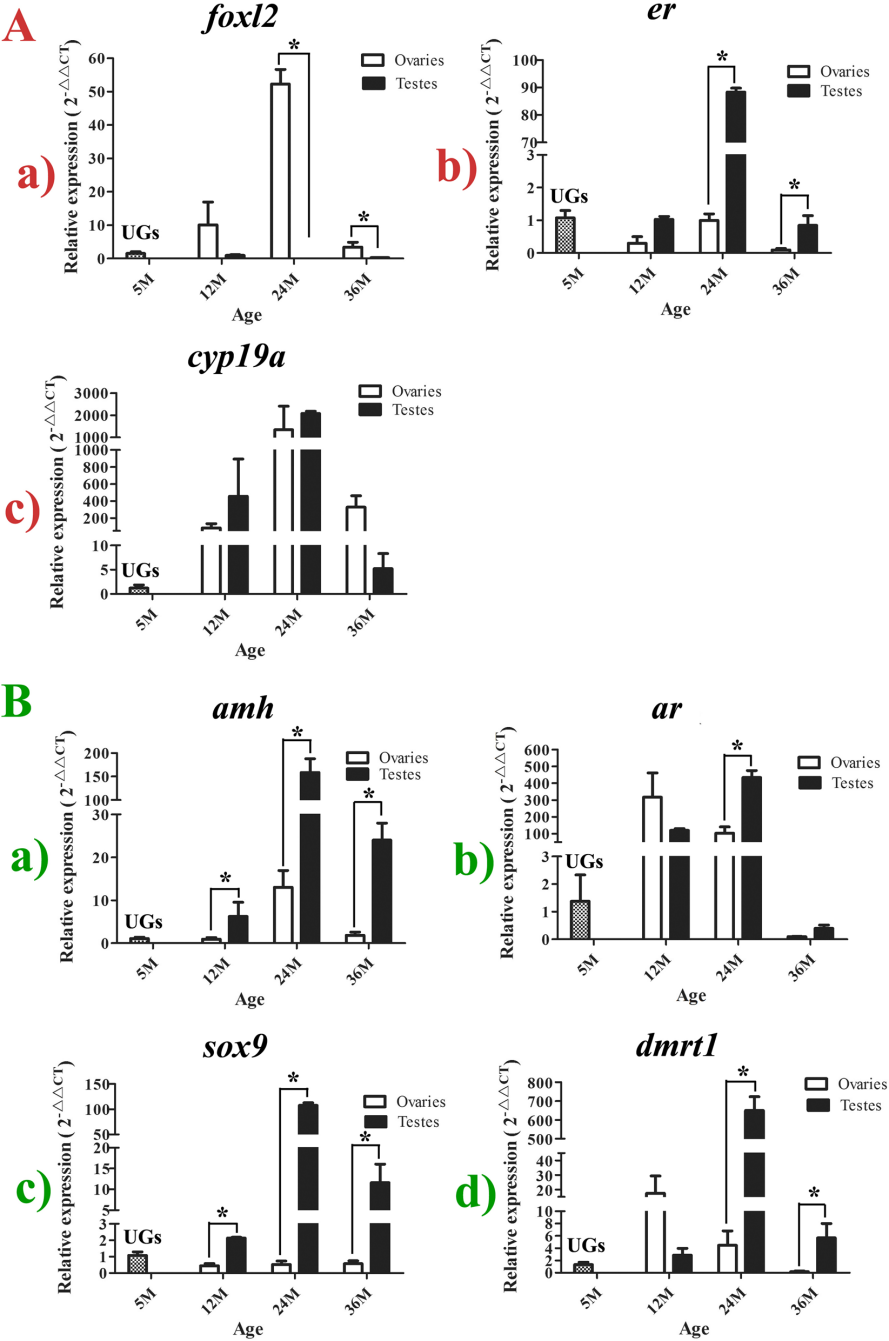
The spatiotemporal expression patterns of seven sex-related genes during sex differentiation of gonads in *A. schrenckii* using real-time PCR. **A** The three ovarian differentiation-related genes, including *foxl2* (Aa), *er* (Ab), and *cyp19a* (Ac). **B** The four testis differentiation-related genes, including *dmrt1* (Ba), *amh* (Bb), *sox9 (Bc)*, and *ar (Bd)*. *Indicates *P* < 0.05 with a statistically significant difference between the two groups

To elucidate the underlying molecular mechanisms, real-time PCR was used for detecting the spatiotemporal expression changes of three ovarian differentiation-related genes (*foxl2*, *er*, and *cyp19a*) and four testis differentiation-related genes (*dmrt1*, *amh*, *sox9*, and *ar*) during the early stages of sex differentiation in *A. schrenckii*. The expression patterns of the ovarian differentiation-related genes during sex differentiation of gonads are shown in Fig. 6A. *Foxl2* was predominantly expressed in the ovaries. The expression levels of *foxl2* were always relatively and significantly higher in the ovaries at 12, 24, and 36 M than in the testes, with the highest expression levels in ovaries at 24 M (*P* < 0.05) (Fig. 6Aa). In contrast to *foxl2*, the transcriptional expression levels of the *er* gene appeared to be the opposite between the sexes. The expression levels of *er* in the testes were higher than that in the ovaries at every stage, the highest value in the testes being observed at 24 M (Fig. 6Ab). The expression characteristics of *cyp19a* changed dramatically between the testes and ovaries during the progression of the sex differentiation process (Fig. 6Ac). First, after extremely low expression in the UGs, the expression level of *cyp19a* was markedly high in both the testes and ovaries. Although the expression levels of *cyp19a* in the testes were slightly higher than that in the ovary at 12 M and 24 M, it was significantly higher in the ovaries than in the testes at 36 M. This result maybe suggests that *cyp19a* only plays an important role in oogenesis in sturgeons.

The expression changes of four testis differentiation-related genes during sex differentiation were also characteristic (Fig. 6B). Amh and *sox9* were relatively consistent in terms of expression patterns, whereas *ar* and *dmrt1* appeared to show a more similar trend. Specifically, both the expression levels of Amh and *sox9* were relatively low in the UGs, and they significantly increased in the testes at all differentiated stages compared to their expression pattern in the ovaries (*P* < 0.05) (Fig. 6Ba,c). These results suggest that *amh* and *sox9* play a significant role in male sex differentiation and that they could be used as early molecular markers in distinguishing males from females. The most remarkable characteristics of *ar* and *dmrt1* were their higher expression in the ovaries than in the testes at 12 M and their significantly higher expression in the testes compared with that in the ovaries at subsequent stages (24 and 36 M) (Fig. 6Bb,d). This result suggests that they are involved in regulating the early sex differentiation process of sturgeons.

## Discussion

In the present study, we explored the role of non-coding miR-34b/c during the early sex differentiation of sturgeons. First, we determined the differentiated features of the histomorphology and germ cells of Amur sturgeons from different developmental stages, ranging from 5 to 36 M. Sturgeons are sexually monomorphic; however, they show no sexually distinguishing morphological characteristics at the larval, juvenile, or even adult stages. The timing of early sex differentiation varies greatly with different sturgeon species and possible aquaculture environments. For instance, juvenile Amur sturgeons (*A. schrenckii*) show different sexual traits in the first six months post-hatching or until nine months [5]. The timing of sex differentiation in *A. schrenckii* is similar to that of the Adriatic sturgeon (*A. naccarii*) [32] and shortnose sturgeon (*A. brevirostrum*) [33]; however, it is earlier than that in the Siberian sturgeon (*A. baerii*) (7 months) [34] and Chinese sturgeon (*A. sinensis*) (9 months). In the present study, individuals belonging to *A. schrenckii* exhibited different male or female traits at 8 months after hatching, with a male/female differentiation ratio of 75.86% (22/29).

Although miR-34b/c has been widely found to play an important role in the progression of various cancers, their expression traits in the gonads of animals attract greater interest. In mice, all previous studies conclusively showed that miR-34b and miR-34c were not expressed in many main tissues (for example, liver, heart, kidney, spleen, and muscle); however, they exhibited exclusive expression in the gonads, and they were specifically abundant in the testis [19–21, 35]. In fish, our previous study indicated that the expression patterns of miR-34b/c in sturgeons were consistent with that in mice [27]. However, a previous study reported that miR-34b expression in zebrafish was enriched in the kidney and olfactory placode [36], which suggests its specific roles in fish, different from its role in mammals. In the present study, miR-34b/c expression during sex differentiation was first uncovered in fish, suggesting its strong relationship with the sex differentiation of sturgeons. Furthermore, miR-34c was localized in the meiotic cells of mice, pachytene spermatocytes, and round spermatids; however, no signal was observed in Sertoli cells and Leydig cells [36]. In sturgeons, the testes showed spermatogonial cell-specific expression of miR-34c and no signal in somatic cells, consistent with the results in mice. In the ovaries, miR-34c was mainly expressed in the cytoplasm of oogonia cells but not in growing oocytes. The relatively low expression of miR-34c in the ovaries of sturgeons is possibly correlated with the maintenance of normal ovarian function because of its abnormally high expression, which induces cell-cycle arrest in an ovarian cancer cell line [24].

miRNAs have been implicated in the regulation of many important biological pathways, such as proliferation, apoptosis, and differentiation. miR-34b/c has been extensively reported as a tumor suppressor that inhibits proliferation and triggers apoptosis in cancer cells, including breast cancer cells [37], lung squamous cell carcinoma [38], and human ovarian cancer cells [39]. In mice, miR-34b/c^−/−^ mutants were specifically found to be correlated with a high incidence of apoptosis in pachytene and elongating spermatids [21]. However, the effect of the miR-34 family on gonad function in fish remains unclear. In the present study, we first established a proliferation system of primary germ cells for a short-term *in vitro* culture, which were isolated from undifferentiated gonads of Amur sturgeon individuals (5 to 7 M). Dabry’s sturgeon germ cells from differentiated stages (22–26 months old) were successfully cultured *in vitro*, as reported previously [40]. A modified one-step digestion method was performed using a three-enzyme mixture in a 25 °C water bath; this method is appropriate for acquiring a single-cell suspension because of the shortened isolation time and increased cell activity. Therefore, we evaluated the effects of miR-34b/c on the proliferation and apoptosis of sturgeon germ cells. The results indicated that miR-34b/c is involved in the fate of undifferentiated sturgeon germ cells, including enhanced cell proliferation and reduced apoptosis. The effects of miR-34b/c on the differentiation fates (i.e., spermatogonial cells or oocyte cells) of germ cells were not evaluated because of the lack of a differentiation culture system for sturgeon germ cells. A previous study showed that miR-34c promotes mouse embryonic stem cell (mESC) differentiation into male germ-like cells [41]. In future investigations, we aim to establish an effective tool and further reveal the effects of miRNAs on regulating the differentiation process of sturgeon germ cells.

During sex differentiation in teleost fishes, there may be a negative feedback regulation mechanism between certain female differentiation-related genes (including *foxl2*, *cyp19a*, and *er*) and male differentiation-related genes (*dmrt1*, *amh*, *cyp11b2*, and *ar*). For example, in genetic XX tilapia, the binding of *foxl2* to the promoter region of cyp19a (cytochrome P450 1A) plays a decisive role in ovarian differentiation by regulating *cyp19a* expression and possibly the entire steroidogenic pathway [42]. Conversely, transgenic overexpression of *dmrt1* results in decreased *cyp19a* expression and serum estradiol-17 beta levels, and knock-out of *foxl2* is accompanied by a high expression of *dmrt1* and *cyp11b2*, causing complete female-to-male sex reversal in genetic XX fish [43, 44]. In the course of androgen-induced masculinization in rainbow trout (*O. mykiss*) and grouper (*Epinephelus sp. coioides*), similar expression networks were found, including up-regulated expression of *dmrt1* and *cyp11b2* and inhibited expression of *foxl2* and *cyp19a*, which promoted gonadal fate toward the testis [45, 46]. Meanwhile, feminization induced by treatment with the anti-androgen flutamide and estrogen revealed that the two treatment groups shared common and similar gene expression patterns, including down-regulation of *ar* and *er* expression and up-regulation of *amh* and *dmrt1* expression [47]. In the present study, we observed that *foxl2* expression in the ovaries and *amh* and *sox9* expression in the testes were negatively regulated by feedback. *Foxl2* could be a marker of early ovary differentiation in Amur sturgeons, which is consistent with the results of a previous study [5]. Meanwhile, *amh* and *sox9* could be markers of early testis differentiation in Amur sturgeons. Dmrt1 does not appear to play a role in early sex differentiation in *A. schrenckii*, which is different from its role of sex determination in mammals [48], birds [49] and certain teleost fishes (*O. latipes* and *O. curvinotus*) [50, 51]. Furthermore, the expression of the two testis differentiation-related genes *ar* and *dmrt1* were higher at 12 M ovaries than testes. The similar expression patterns of *ar* were also found in sexually mature *A. ruthenus* [52] and 21 M Best Beluga sturgeon [53]. Therefore, we speculate that the abnormal expression patterns of *ar* and *dmrt1* in gonads may be related to development stage or species in sturgeon.

**Fig. 7:**
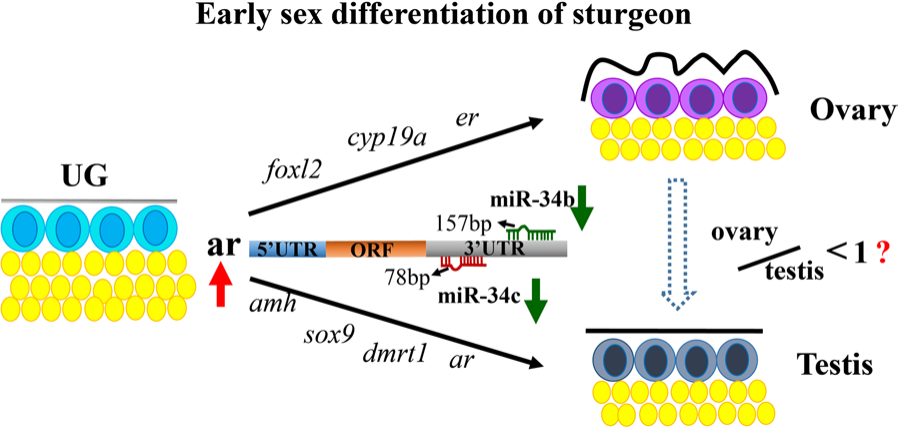
Proposed model for direct targeting of the *ar* pathway by miR-34b/c in early sex differentiation of sturgeons. In the model, miR-34b/c interacts with female differentiation-related genes (including *foxl2*, *cyp19a*, and *er*) and male differentiation-related genes (*dmrt1*, *amh*, *cyp11b2*, and *ar*) via *ar*. Possibly, inhibition of miR-34b/c (down-regulation) and overexpression of *ar* (up-regulation) reduce the ovary/testis differentiation ratio, i.e., greater male-fate differentiation

In both human and mouse models, miR-34b/c plays a crucial role in normal testicular function, as well as in successful spermatogenesis, regulating spermatozoa maturation and functionality. Previous studies have demonstrated that miR-34b/c targets multiple genes. For example, at the initial stages of spermatogenesis, miR-34b/c targets several transcripts of cell-cycle regulatory proteins, including *Cdks*, *cyclins*, *E2F-pRB*, and *Myc*, leading germ cells to exit the cell cycle for initiation of spermatogenesis [54]. miR-34c promotes mESC differentiation into male germ-like cells via the retinoic acid receptor gamma (*RARg*) gene [41]. miR-34b/c normally regulates the stability of *Ccdc113* and *Dnah6*, which are involved in multiciliogenesis, to ensure the proper development of motile cilia of the male reproductive system [55]. The existence of several target genes for miR-34b/c is unsurprising, because it is commonly accepted that one miRNA can target numerous mRNAs, and one particular mRNA can be targeted by multiple miRNAs [56]. Additionally, sex-biased expression of miRNAs may directly control the differential expression of many target genes that contribute to different sexual traits during sex differentiation. In the present study, luciferase activities were significantly changed, suggesting that *ar* is the true target of miR-34b/c in sturgeons. *Ar* exhibited high expression levels in both the ovaries and testes during early sex differentiation in sturgeons. Importantly, we observed that the expression patterns of miR-34b/c and *ar* were negative during the sex differentiation of sturgeons. *Ar* is expressed in Sertoli cells, oocytes, granulosa cells, and theca cells, and it has been verified to be important for normal testis function [57] and ovary development [58]. *Ar* has been reported to play a key role in sex determination and differentiation. For example, *ar*-mutant zebrafish exhibit a female-biased sex ratio relative to wild-type controls, suggesting that *ar* plays a role in promoting male determination [59, 60]. Meanwhile, in the Japanese frog, *ar*-Tg ZW frogs (ZW (genetic female) zygote transferred exogenous *ar*) revealed development masculinized gonads or ‘ovotestes’, which indicates that *ar* is involved in male sex determination in an amphibian species [61]. Therefore, we conclude that miR-34b/c directly targets the *ar* pathway and expression regulation network with female differentiation-related genes (including *foxl2, cyp19a*, and *er*) and male differentiation-related genes (*dmrt1*, *amh*, *cyp11b2*, and *ar*), and that it is involved in early sex differentiation of sturgeons (Fig. 7), which may validate the hypothesis regarding multi-gene regulation mechanisms of sex determination and differentiation in sturgeons [62]. Furthermore, the inhibition of miR-34b/c induces up-regulation of *ar* and causes further male-fate differentiation, resulting in an ovary/testis differentiation ratio less than 1. Therefore, the role of miR-34b/c and *ar* interaction in sex determination and differentiation of sturgeons may be further elucidated by studying the effect of gonadal injections of miR-34b/c mimics and inhibitors in animals.

## Conclusion

In summary, our results showed that miR-34b/c is a key miRNA involved in the regulation of early sex differentiation in sturgeons. miR-34c was mainly expressed in the cytoplasm of oogonia cells in the ovary and the nucleus of spermatogonial cells in the testis. Quantitative real-time PCR indicated that miR-34b/c showed similar spatiotemporal expression patterns and significantly increased during early sex differentiation. The overexpression and inhibition of miR-34b/c suggested its effects on the biological process of germ cells in sturgeons, including enhancing the proliferation ability of germ cells and reducing apoptosis. Luciferase reporter assays verified the targeted regulatory interaction between miR-34b/c and the 3′-UTR of *ar* mRNA.

## Supplementary Information

**Additional file 1: Table S1**. Growth performance of Amur sturgeon individuals.

**Additional file 2: Table S2**. Stem-loop RT primers and amplification primers were used for real-time PCR.

**Additional file 3: Table S3**. Germ-cell-specific gene primers used by RT-PCR.

**Additional file 4: Table S4**. Primers information for 3'UTR region of ar containing Xho I and Not I restriction sites.

**Additional file 5: Fig. S1**. Morphological observation of the gonads at the sex-undifferentiated stage. **A**, **B** show gonads at 5 M. **C**,** D **present gonads at 6 M. The sections of the gonads are cross (**A** and **C**) and longitudinal (**B** and **D**). Thick fat and smaller volume gonads were at 5 M, and the volumes of the gonads at 6 M were significantly growing compared with that at 5 M. F, fat; G, undifferentiated gonads. The gonadal tissues were stained with HE staining. Scale bar = 100 μm.

**Additional file 6: Fig. S2**. *In vitro* culture of germ cells of Amur sturgeon from the sensitive stage of early sex differentiation. **A** Single cells isolated from gonads by a one-step method, using a three-enzyme-mixture digestion method. **B** *In vitro* 48 h, germ cells aggregated on the feeding layer into three-dimensional (3D) suspension growth. **C** *In vitro* four days, germ cells began to proliferate, and the numbers increased significantly. **D** At seven days *in vitro*, germ cells proliferated into larger clonal clusters. Scale bar = 50 μm. **E** Undifferentiated germ-cell-specific markers were detected to be expressed in cultured germ cells *in vitro*. The positive controls were the ovary and testis obtained from Amur sturgeon individuals (36 M).

**Additional file 7: Fig. S3**. The construction of dual-luciferase reporter system for *ar*-3’UTR-psiCHECK-2 luciferase vector. **A** Gel electrophoresis of Xho I和Not I double enzyme digestion. 1 and 2 represent two different clone duplications. **B** The sequence identification of *ar* 3’UTR from the monoclonal bacterial solution PCR of *ar*-3’UTR-psiCHECK-2 luciferase vectors. Seven clone bacterial solutions were randomly chosen. Marker2000 was used, and 381 bp of *ar* was the target fragment..

## Data Availability

All data generated or analyzed during this study included in the published article and its additional files.
