# Supplementary figures and images for: MiR-34b/c play a role in early sex differentiation of Amur sturgeon, Acipenser schrenckii
Source: Front Zool. 2022 Sep 26;19:23. doi: 10.1186/s12983-022-00469-6 (PMC9511750; doi:10.1186/s12983-022-00469-6)

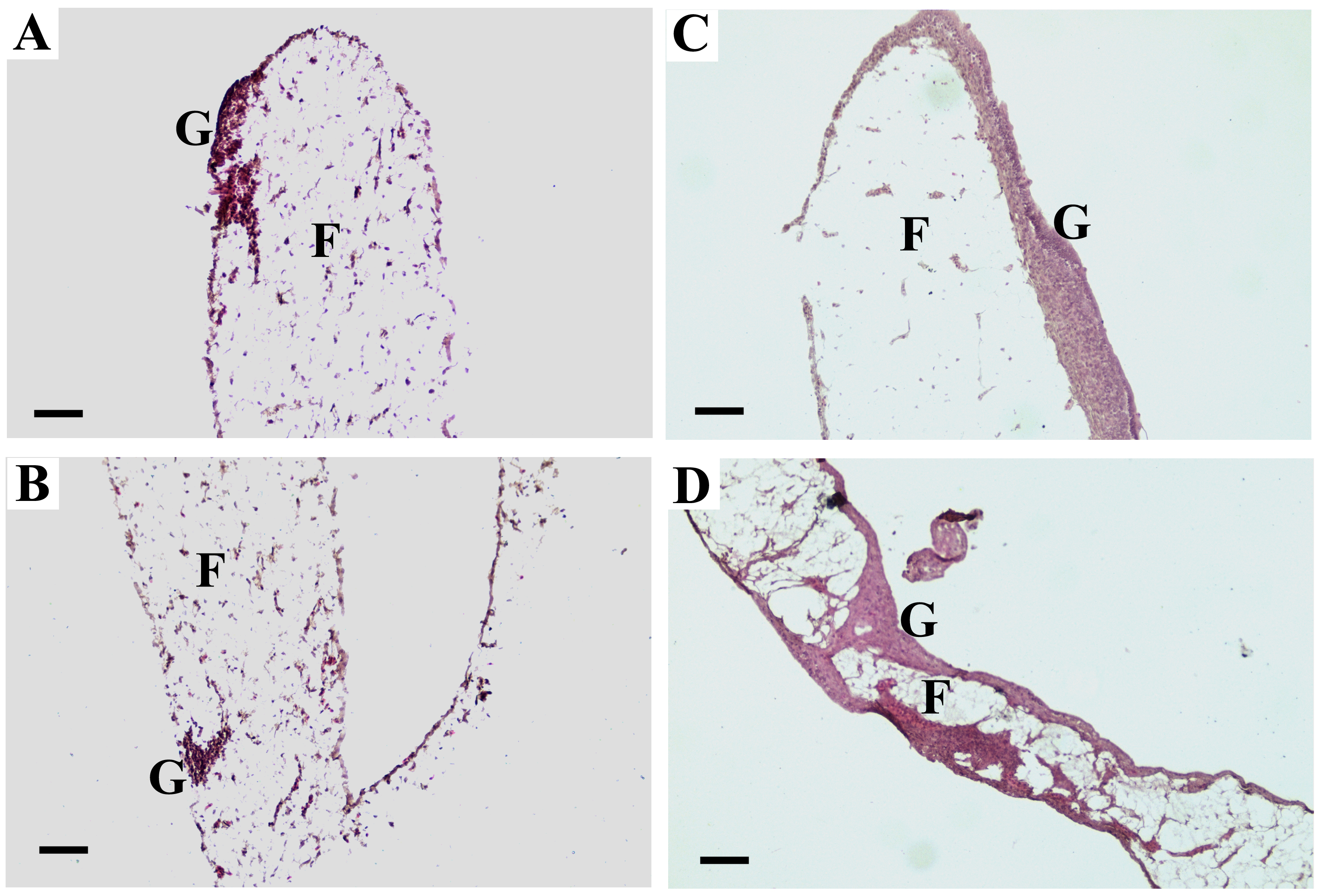

Supplement: Supplementary file 5 — Additional file 5: Fig. S1. Morphological observation of the gonads at the sex-undifferentiated stage. A, B show gonads at 5 M. C, D present gonads at 6 M. The sections of the gonads are cross (A and C) and longitudinal (B and D). Thick fat and smaller volume gonads were at 5 M, and the volumes of the gonads at 6 M were significantly growing compared with that at 5 M. F, fat; G, undifferentiated gonads. The gonadal tissues were stained with HE staining. Scale bar = 100 μm. [file 12983_2022_469_MOESM5_ESM.jpg]

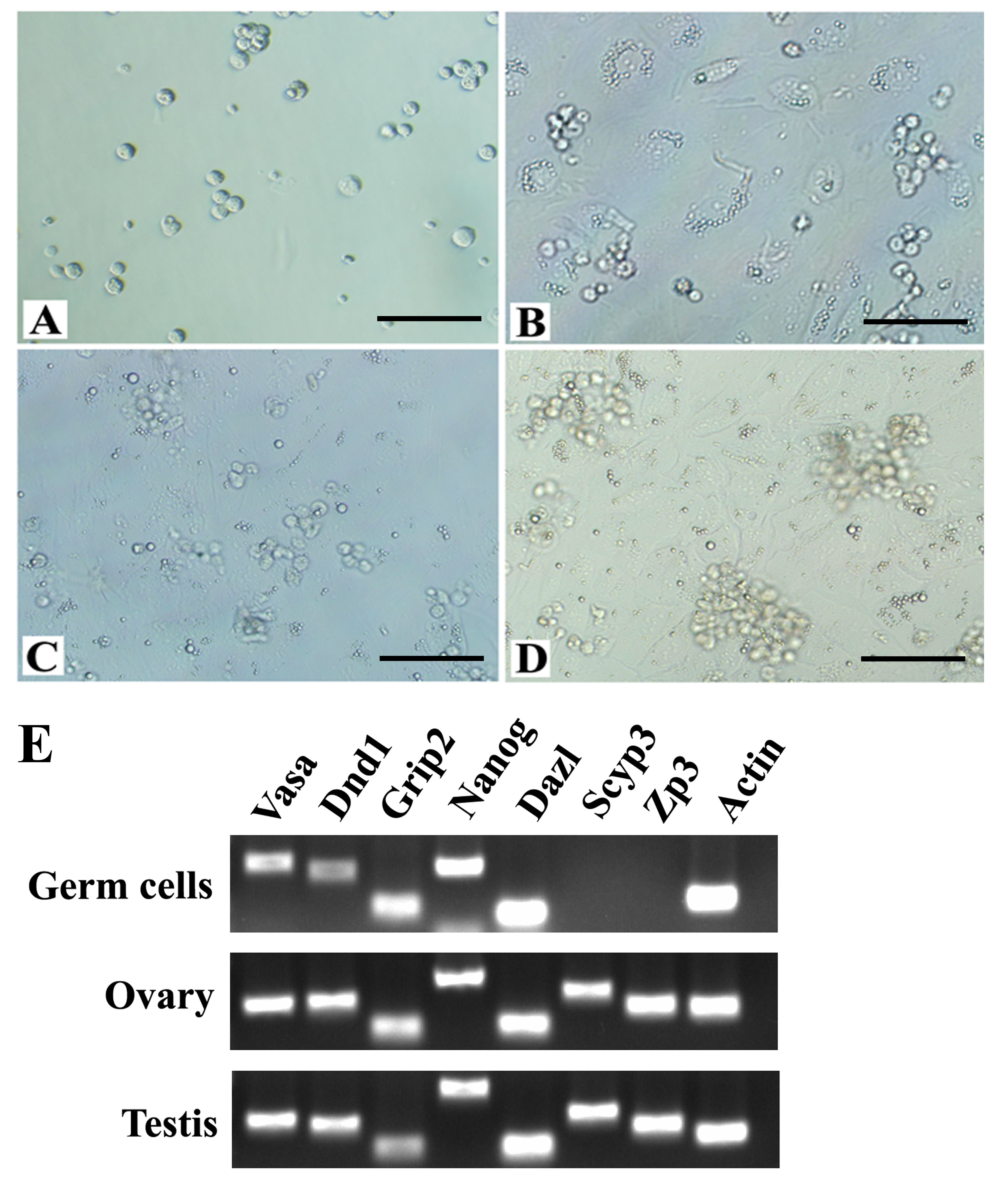

Supplement: Supplementary file 6 — Additional file 6: Fig. S2. In vitro culture of germ cells of Amur sturgeon from the sensitive stage of early sex differentiation. A Single cells isolated from gonads by a one-step method, using a three-enzyme-mixture digestion method. B In vitro 48 h, germ cells aggregated on the feeding layer into three-dimensional (3D) suspension growth. C In vitro four days, germ cells began to proliferate, and the numbers increased significantly. D At seven days in vitro, germ cells proliferated into larger clonal clusters. Scale bar = 50 μm. E Undifferentiated germ-cell-specific markers were detected to be expressed in cultured germ cells in vitro. The positive controls were the ovary and testis obtained from Amur sturgeon individuals (36 M). [file 12983_2022_469_MOESM6_ESM.jpg]

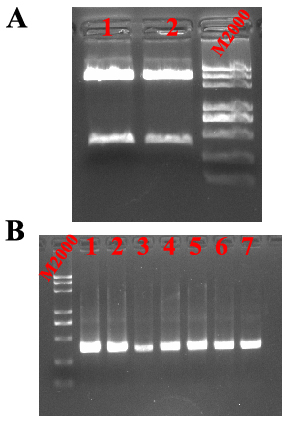

Supplement: Supplementary file 7 — Additional file 7: Fig. S3. The construction of dual-luciferase reporter system for ar-3’UTR-psiCHECK-2 luciferase vector. A Gel electrophoresis of Xho I和Not I double enzyme digestion. 1 and 2 represent two different clone duplications. B The sequence identification of ar 3’UTR from the monoclonal bacterial solution PCR of ar-3’UTR-psiCHECK-2 luciferase vectors. Seven clone bacterial solutions were randomly chosen. Marker2000 was used, and 381 bp of ar was the target fragment.. [file 12983_2022_469_MOESM7_ESM.jpg]
